# Risk factors for reoperation after flexor tendon repair: a registry study

**DOI:** 10.1177/17531934221101563

**Published:** 2022-05-17

**Authors:** Jonas Svingen, Monica Wiig, Christina Turesson, Simon Farnebo, Marianne Arner

**Affiliations:** 1 Karolinska Institutet, Department of Clinical Science and Education, Södersjukhuset, Stockholm, Sweden; 2 Department of Hand Surgery, Södersjukhuset, Stockholm, Sweden; 3 Department of Surgical Science, Hand surgery, Uppsala University, and Uppsala University Hospital, Sweden; 4 Department of Hand Surgery, Plastic Surgery and Burns, and Department of Biomedical and Clinical Sciences, Linköping University, Linköping, Sweden; 5 Department of Health, Medicine and Caring Sciences, Division of Prevention, Rehabilitation and Community Medicine, Linköping University, Linköping, Sweden

**Keywords:** Risk factors, tendon rupture, complication, tenolysis, flexor tendon

## Abstract

The aim of this study was to identify risk factors for reoperations after Zones 1 and 2 flexor tendon repairs. A multiple logistic regression model was used to identify risk factors from data collected via the Swedish national health care registry for hand surgery (HAKIR). The studied potential risk factors were age and gender, socio-economics and surgical techniques. Included were 1372 patients with injuries to 1585 fingers and follow-up of at least 12 months (median 37 IQR 27–56). Tendon ruptures occurred in 80 fingers and tenolysis was required in 76 fingers. Variables that affected the risk of rupture were age >25 years (*p* < 0.001), flexor pollicis longus tendon injuries (*p* < 0.001) and being male (*p *= 0.004). Injury to both finger flexors had an effect on both rupture (*p* = 0.005) and tenolysis (*p* < 0.001). Understanding the risk factors may provide important guidance both to surgeons and therapists when treating patients with flexor tendon injuries.

**Level of evidence:** III.

## Introduction

Flexor tendon repair is a common and much studied procedure in hand surgery. Despite substantial improvements of results over the last decades, reoperation rates of between 6% (Dy et al., 2012a) and 13% (Rigo and Røkkum, 2016) have been reported. Tendon ruptures and adhesion formations are the most frequent reasons for reoperation after flexor tendon repair, with a reported frequency of around 4%, respectively, for both complications (Dy et al., 2012b).

Much remains unknown regarding factors that may increase the risk for a reoperation after flexor tendon repair. In particular, there is a lack of studies with large cohorts of patients for the comparison of different factors, including socioeconomic variables and detailed descriptions of the surgical procedure. The aim of this study was to identify factors affecting the frequency of reoperations due to tendon rupture or adhesions, in a large cohort of patients following Zones 1 and 2 flexor tendon repairs.

## Methods

### Data retrieval

The Swedish national health care registry for hand surgery (HAKIR) (Arner, 2016) collects information on patients who underwent specialized hand surgery in Sweden, and was used for data collection in this study. Data on all patients operated between October 2010 and December 2018 with primary repair of a complete finger or thumb flexor tendon injury in Zones 1 and 2, were collected from HAKIR. Exclusion criteria included patients with concomitant fractures and extensor tendon injuries. The study was approved by the regional ethics board (Dnr 2017/2023-31 and 2018/1106-32, Stockholm, Sweden).

For the same cohort, registry data regarding reoperations performed from October 2010 to December 2019 was collected from HAKIR. The interval was chosen to allow for at least 12 months of follow-up. The reoperations were divided into two separate outcome categories as defined by the primary reason for reoperation, either re-repair for tendon rupture or tenolysis. We also included socioeconomic data, level of income and education for the included patients from the year before the primary surgery from Statistics Sweden (SCB), the organization responsible for coordinating official statistics in Sweden on assignment from the government.

### Data analysis

Variables that were selected as potential risk factors for reoperation were age, sex, income, level for education, circumstances of injury, for example time between injury and repair, injured hand/tendon, number of injured fingers or concomitant injured nerves, repair techniques, for example core suture techniques/number/circumference.

Core suture material was categorized as braided polyester, non-resorbable monofilament, resorbable monofilament and braided polyblend. Core suture technique was categorized as modified Kessler, loop suture (mainly Tsuge), distal reinsertion to bone, criss-cross and others (mainly Lim-Tsai, Kirchmeyer and mattress suture techniques). Core suture circumference was categorized as 3-0, 4-0 and others (2-0 and 5-0). Core suture number was categorized as 2, 4 and others (5 and 6). All variables were grouped into categories based on clinical relevance and size. Categories with a case count of less than 25 were listed into a subcategory labelled ‘other’.

Income was defined as low (disposable income per consumption unit below 60% of median income for all), middle (between low and high definition) and high (above double the median income). Education was defined as low (pre-high school), middle (high school) and high (post-high school). Level of income and education was based on data from the year before the primary surgery.

All surgeries were performed or supervised by an experienced hand surgeon at one of the specialized hand surgery departments in Sweden. All patients received a dorsal cast or a splint to wear during the first 4 to 6 weeks (mean 29 days, SD 9.5) with the metacarpophalangeal (MCP) joints in flexion. The patients received written and oral instruction not to use the injured hand during the initial restriction period. Information on the type of rehabilitation protocol was missing in 801 patients (57%). Of the 595 patients with complete rehabilitation data, 440 (74%) had early active motion, 126 (21%) had early passive motion with a Kleinert device, 14 (2.4%) had active hold, 14 (2.4%) had cast immobilization and one patient had the Manchester short splint. All patients were followed-up with regular appointments by a physiotherapist or an occupational therapist specialized in treating flexor tendon injuries during the rehabilitation period.

### Statistical analysis

Data were check for normal distribution with the Shapiro–Wilk test. Non-normal distributed data is reported in median and interquartile range (IQR), and normal distributed data as mean and standard deviation (SD). Logistic regression was used to examine the associations between the selected variables and the outcomes. This was done in several steps, first as single predictor logistic regression to examine the unadjusted association by odds ratios of each variable to the need for reoperation, because of rupture or adhesions. Second, we used multivariable models to examine the adjusted associations between variables and outcome (Model 1). Finally, multivariable models were used with only significant variables *p* < 0.05 from the unadjusted model and Model 1 (Model 2). After adding significant variables to Model 2, variables with missing values >10% and *p* > 0.05 were removed. The potential interaction effect between variables was tested with significant variables from Model 2, the interaction was considered significant if *p* < 0.05. Additional information about the assessment of missing data, multiple observations and assumptions of logistic regression are as shown in the Supplementary online Appendix S1.

## Results

After exclusion of 24 fingers with concomitant fracture and two with extensor tendon injuries, the final study sample included a total of 1372 patients with injury to 1585 fingers with a median age of 33 years (IQR 23–47), of which 29% were women.

There was a median of 37 months (IQR 27–56) between primary repair and the end of follow-up. There was a total reoperation rate of 9.8% in the 1585 fingers. Reoperation for re-repair of the tendon due to tendon rupture was performed in 80 fingers and tenolysis was performed in 76 fingers, leaving a total rupture rate of 5% and a tenolysis rate of 4.8%. Median number of days to reoperation from the primary surgery was 25 days (IQR 14–52) in the fingers that presented with rupture, and 307 days (IQR 239–455) in the tenolysis group. Twenty-three (29%) of the fingers with tendon rupture and six (7.9%) of the fingers reoperated with tenolysis needed further surgery. Five categories had missing data >10% (Table S1 and S2). The partial missing data in variables meant that the total missing cases was 52% in the first multivariable model for both outcomes, leaving a total of 764 complete cases. In the final multivariable model (Model 2), missing cases were 3.2% for the rupture outcome and 5.3% for the tenolysis outcome.

### Analysis of associations with tendon rupture

Patients’ sex, age and type of tendon injury had an association with the risk of tendon rupture (*p* < 0.05), in both the crude measures and multivariable models (Tables 1 and S3). Patients in the age groups 25–50 years and older than 50 years had a higher association with a risk of rupture 6.4% and 7.1%, respectively, as compared with patients in the younger age group (1.3%) (*p* < 0.001) (Table S3). This corresponded to an odds ratio (OR) 5.5 in patients older than 50 years (Table 1). Men also had a higher association with tendon rupture, 6.1%, as compared with women, 2.3% (*p* < 0.004). There was an interaction effect between age and sex on the risk of rupture. Men in the age group 25–50 years had a rupture rate of 8.7% compared with 0.9% in women, in the same age group (Table 2).

**Table 1.** Information regarding the individual variables and their adjusted associations to rupture after flexor tendon repair in Zones 1 and 2 in 1585 fingers.

	Model 1 Adjusted for all variables		Model 2 Adjusted for sex, age and injured tendon	
Variables	OR (CI 95%)	*p*-value	OR (CI 95%)	*p*-value
Sex				
Women	Reference		Reference	
Men	3.5 (1.3–9.0)	0.01	2.7 (1.4–5.4)	0.004
Age				
<25	Reference		Reference	
25–50	6.5 (1.5–28.6)	0.014	5.6 (2.4–13.2)	<0.001
>50	10.7 (2.3–50.1)	0.003	5.5 (1.4–5.4)	<0.001
Injured tendon				
FDP	Reference		Reference	
FDP + partial FDS	1.8 (0.6–5.4)	0.275	1.3 (0.6–3.1)	0.492
FDP + FDS	3.5 (1.4–8.4)	0.006	2.4 (1.3–4.4)	0.005
FPL	4.6 (1.1–19.9)	0.041	3.8 (1.9–7.3)	<0.001

OR: odds ratio; CI: confidence interval; FDP: flexor digitorum profundus; FDS: flexor digitorum superficialis; FPL: flexor pollicis longus.

**Table 2.** Interaction between sex and age on the adjusted association to rupture after flexor tendon repair in Zones 1 and 2.

Sex	Age group	Number of fingers (% with rupture)	Adjusted for type of tendon injury^a^OR (CI 95%)	*p*-value
Women	<25	106 (2)	Reference	
	25–50	232 (1)	0.5 (0.1–3.3)	0.445
	>50	83 (7)	3.8 (0.7–19.4)	0.112
Men	<25	357 (1)	0.6 (0.1–3.2)	0.528
	25–50	527 (9)	4.9 (1.2–20.6)	0.030
	>50	206 (7)	3.5 (0.8–15.6)	0.104

^a^Tendon injury type categorized as, flexor digitorum profundus, or flexor digitorum profundus and partial or complete flexor digitorum superficialis, or flexor pollicis longus.

OR: odds ratio; CI: confidence interval.

More severe tendon injuries, involving both the flexor digitorum profundus (FDP) and flexor digitorum superficialis (FDS) tendons, had a higher association with an increased rupture rate, 6.6% compared with 2.7% when only the FDP tendon was injured (*p* = 0.005). Injury to the flexor pollicis longus (FPL) tendon also had a higher association with tendon rupture, 10%, as compared with 2.7%, in FDP injuries (*p* < 0.001). The core suture technique with modified Kessler had an association to rupture in Model 1 as compared with loop suture (*p* = 0.037). When adding core suture technique to Model 2, the variable had no association (*p* = 0.190) and because of the missing cases (18%) and no explanatory effect on the outcome variable, we removed this variable from Model 2, as displayed in Table 1.

### Analysis of associations with tenolysis

The type of tendon injury had an association with the risk of tenolysis (*p* =  0.001) in both the crude and the multivariable measures (Table 3 and Table S3). Patients with injury to both the FDP and FDS tendon had an increased risk of needing tenolysis (7.2%) as compared with patients who only had an FDP tendon injury (3.1%). Patient age (*p* = 0.032) and number of fingers (*p* = 0.033) injured had an association with tenolysis in the first multivariable model. However, there were no associations between these variables and tenolysis in Model 2, or as crude measures. No patients in the group with low income had been reoperated with tenolysis compared with a rate of patients with middle income of 5.4% and with high income of 8.7% (Table S1). There were only a significant association between income and tenolysis in Model 1 (*p* = 0.036), but the incidence of zero tenolysis in the low-income group makes the OR incomparable despite the high probability of a significant association.

**Table 3.** Information regarding the individual variables and their adjusted associations to tenolysis after flexor tendon repair in Zones 1 and 2 in 1585 fingers.

	Model 1 Adjusted for all variables		Model 2 Adjusted for age, income, injured tendon, number of fingers	
Variables	OR (CI 95%)	*p*-value	OR (CI 95%)	*p*-value
Age				
<25	Reference		Reference	
25–50	0.3 (0.1–0.9)	0.032	1.0 (0.6–1.8)	0.965
>50	1.5 (0.6–3.9)	0.425	1.3 (0.6–2.5)	0.509
Income^a^				
Low	n.a	0.996	n.a	0.994
Middle	0.4 (0.4–0.9)	0.036	0.7 (0.3–1.4)	0.307
High	Reference		Reference	
Injured tendon				
FDP	Reference		Reference	
FDP + partial FDS	0.9 (0.3–2.6)	0.848	1.2 (0.9–4.1)	0.057
FDP + FDS	1.6 (0.7–3.7)	0.277	2.7 (1.5–4.9)	0.001
FPL	0.2 (0.0–0.1.0)	0.046	0.9 (0.4–2.4)	0.877
Number of fingers				
Single	9.9 (1.2–81.5)	0.033	0.8 (0.5–1.4)	0.525
Multiple	Reference		Reference	

^a^Low income: disposable income per consumption unit below 60% of median income for all. Middle income: income between low and high definition. High income: above double the median income.

OR: odds ratio; CI: confidence interval; n.a: not applicable, due to zero observation with tenolysis; FDP: flexor digitorum profundus; FDS: flexor digitorum superficialis; FPL: flexor pollicis longus.

## Discussion

In this large registry study, we identified male sex, age above 25 years, injury to both FDP and FDS tendons, and the FPL tendon as significant risk factors for developing a tendon rupture after repair. Tendon injury that included both FDP and FDS tendon was the only variables associated with reoperation with tenolysis. In the low-income group, no patients underwent tenolysis as compared with 5.4% in the middle-income group and 8.7% in the high-income group.

This finding indicates either that patients with better economic conditions are more prone to proceed with a reoperation such as a tenolysis, or that the level of adhesion development was associated with their income. Despite the fact that the Swedish welfare system provides comparably good compensation for all citizens, there seems to be an effect of income on the patients’ willingness, or demand for further treatment, after the initial treatment period is over.

As previous research suggests, we also found that patient age affected the risk of tendon rupture (Dy et al., 2012a; Hurley et al., 2019). An animal study on mice showed impaired tendon healing with increased age, explained by a dramatically less bridging tendon collagen at the repair site with increasing age (Ackerman et al., 2017). We also found an interaction between sex and age. Middle-aged men had an 8.7% rupture rate as compared with 0.9% in women in the same age group. Our results added to the increasing number of publications that suggested male sex as a risk factor for flexor tendon rupture. Recent research have identified the male sex to increase the risk for reoperation due to any type of complication after flexor tendon repair (Lalchandani et al., 2021). Harris et al. (1999) reported higher rupture rates in males although this was not statistically significant. A higher splint removal rate during postoperative rehabilitation had been reported in males (Sandford et al., 2008). This behaviour could increase the risk for tendon rupture and may be an indication for addressing the importance of adherence to rehabilitation programmes in some male patients.

Injury to the FPL tendon had a high rupture rate (10%) and an association with higher OR as compared with the other flexor tendons, but no increased risk for tenolysis. There are a few reports on FPL injuries as compared with injuries to the other flexor tendons, although some inconsistencies exist in the reported rupture rates. Bruin et al. (2020) reported a rupture rate of 5%, and Sirotakova and Elliot (1999) reported rupture rates of 17% for FPL. In contrast, Kasashima et al. (2002) reported zero ruptures in 29 FPL repairs. The increased risk for ruptures of the FPL tendon may possibly be caused by the higher power output and difference in the movement arm compared with the flexor tendons in other fingers during normal grasping activities of the hand (Goodman and Choueka, 2005; Lee and Jung, 2020).

Injury to both the FDS and FDP tendon had an association with both tenolysis and rupture as compared with injury only involving the FDP. High tenolysis rates in injuries to both tendons have previously been reported (Civan et al., 2020). Zone 2 injuries have been associated with a lower range of motion (Rigo and Røkkum, 2016) and are well established as the most difficult area of suture, although there are a lack of studies comparing different zones (Elliot and Giesen, 2013; Hurley et al., 2019).

There is conflicting evidence regarding the optimal suture technique to avoid tendon adhesions and ruptures. In our study, we found a significantly higher operation rate for rupture with the modified Kessler techniques, as compared with loop suture techniques, in the first multivariable model but not in the second model. The high percentage of missing data in the first model could have had an effect on the results and when combining the results from the other models, we interpreted the variable as not being a risk factor. The transverse component of modified Kessler repair has been shown to have a negative effect on the tensile resistance of 4-strand tendon repairs, with increasing risk for gapping upon load (Wu et al., 2021). A meta-analysis, however, showed that the core suture technique did not influence the rupture rate (Dy et al., 2012b).

There are limitations in our study. First, there is partially missing data, especially regarding the surgical variables. To assess the impact of this in Model 1, we compared frequency of rupture and tenolysis between the unadjusted model and Model 1. We also compared the frequency of cases within each subcategory between the models. The differences within subcategories were <5% except for repairs with the modified Kessler technique, which may have contributed in the association to rupture in Model 1. Second, the retrospective nature of our study meant that certain information is lacking on some potential risk factors, such as smoking or the injury mechanism. In the present study, we also did not include potential risk factors that may occur after surgery, such as poor adherence to rehabilitation and the effect of more active rehabilitation regimes; factors that have previously been linked to higher rupture rates (Harris et al., 1999). The data on rehabilitation regimes in HAKIR is collected at the 3-month follow-up for functional assessments. Fourth, information about the circumferential suture was not included in our data, which may be considered when interpretating the results. Finally, we did not include information on the surgeons who operated on these injuries, although as is customary in Sweden, all flexor tendons would have been referred to, and operated by (or supervised) by an experienced hand surgeon at one of the specialized hand surgery departments.

In conclusion, this is the first large study on reoperations after flexor tendon repair including detailed variables on surgical techniques as well as socioeconomic data. We identified several risk factors for reoperation after finger flexor surgery. Understanding these risk factors may give important guidance both to surgeons and therapists when treating patients with flexor tendon injuries. Future research should consider explanatory variables such as rehabilitation method, smoking and injury mechanism.

## Supplemental Material

sj-pdf-1-jhs-10.1177_17531934221101563 - Supplemental material for Risk factors for reoperation after flexor tendon repair: a registry study Click here for additional data file. Supplemental material, sj-pdf-1-jhs-10.1177_17531934221101563 for Risk factors for reoperation after flexor tendon repair: a registry study by Jonas Svingen, Monica Wiig, Christina Turesson, Simon Farnebo and Marianne Arner in Journal of Hand Surgery (European Volume)

sj-pdf-2-jhs-10.1177_17531934221101563 - Supplemental material for Risk factors for reoperation after flexor tendon repair: a registry study Click here for additional data file. Supplemental material, sj-pdf-2-jhs-10.1177_17531934221101563 for Risk factors for reoperation after flexor tendon repair: a registry study by Jonas Svingen, Monica Wiig, Christina Turesson, Simon Farnebo and Marianne Arner in Journal of Hand Surgery (European Volume)

sj-pdf-3-jhs-10.1177_17531934221101563 - Supplemental material for Risk factors for reoperation after flexor tendon repair: a registry study Click here for additional data file. Supplemental material, sj-pdf-3-jhs-10.1177_17531934221101563 for Risk factors for reoperation after flexor tendon repair: a registry study by Jonas Svingen, Monica Wiig, Christina Turesson, Simon Farnebo and Marianne Arner in Journal of Hand Surgery (European Volume)

sj-pdf-4-jhs-10.1177_17531934221101563 - Supplemental material for Risk factors for reoperation after flexor tendon repair: a registry study Click here for additional data file. Supplemental material, sj-pdf-4-jhs-10.1177_17531934221101563 for Risk factors for reoperation after flexor tendon repair: a registry study by Jonas Svingen, Monica Wiig, Christina Turesson, Simon Farnebo and Marianne Arner in Journal of Hand Surgery (European Volume)
