# Supplementary material for: Risk factors for reoperation after flexor tendon repair: a registry study
Source: J Hand Surg Eur Vol. 2022 May 17;47(10):1071–6. doi: 10.1177/17531934221101563 (PMC9634328; doi:10.1177/17531934221101563)
Supplement: sj-pdf-1-jhs-10.1177_17531934221101563 - Supplemental material for Risk factors for reoperation after flexor tendon repair: a registry study [file sj-pdf-1-jhs-10.1177_17531934221101563.pdf]

Supplementary statistical analysis

The Wald test was used to test the significance of each variable at a level of $p<0.05$. Associations are shown as odds ratios (OR) with confidence intervals (CI) at 95 %. Cases with missing data in any variables were removed from the multivariable models. Hosmer Lemeshow test was used to test the goodness-of-fit in the last model. Influential observations were checked by plotting Cook´s distance with a cut off >1. Nested models with same observations were tested with -2 log likehood and comparing Nagelkerke R square. The influence of multiple observations was assessed by comparing Cohen´s kappa between variables, between patients with multiple and individual observations.

The differences within subcategories were <5% except for modified Kessler which may have contributed in the association to rupture in model 1. The two sub - variables; FPL, and digit 1 injury violated the assumption of independent observation because these were identical. This led to an exclusion of digit 1 injuries in model 1. The Cohen´s kappa between patients with single and multiple observation was <0.2 with the highest observed value 0.254 (considered fair) for patients with multiple observations (Gisev et al., 2013). This indicates that the amount of clustering as a result of multiple observations from the same patient, was relatively low. Hosmer Lemeshow indicated good fit of model (p=0.935). For the analysis of rupture the 2loglikehood test and Negelkerke R suare indicated that the model with interactions was better compared to the model without interaction. Cook´s distance showed no influential observation >1. For the analysis of tenolysis Hosmer Lemeshow indicated good fit of model (p=0.381). Cook´s distance showed no influential observations >1.
