# Supplementary material for: Risk factors for reoperation after flexor tendon repair: a registry study
Source: J Hand Surg Eur Vol. 2022 May 17;47(10):1071–6. doi: 10.1177/17531934221101563 (PMC9634328; doi:10.1177/17531934221101563)
Supplement: sj-pdf-2-jhs-10.1177_17531934221101563 - Supplemental material for Risk factors for reoperation after flexor tendon repair: a registry study [file sj-pdf-2-jhs-10.1177_17531934221101563.pdf]

Supplementary table 1. Demographic of variables of patients and injury in the study cohort. Rate of rupture and tenolysis within each variable.

Variable	Total number of patients (% of cohort)	Total number of fingers (% of cohort)	Rupture rate (% fingers in variable)	Tenolysis rate (% fingers in variable)
**Sex**				
Women	392 (28.6)	429 (27)	10 (2.3)	25 (5.8)
Men	980 (71.4)	1156 (73)	70 (6.1)	51 (4.4)
**Age**				
< 25	379 (27.6)	463 (29.2)	6 (1.3)	20 (4.3)
25 - 50	707 (51.5)	811 (51.2)	52 (6.4)	38 (4.7)
> 50	286 (20.8)	311 (19.6)	22 (7.1)	18 (5.8)
**Income**[a]				
low	195 (14.2)	223 (14.1)	13 (5.8)	0 (0.0)
middle	1043 (76)	1213 (76.5)	61 (5.0)	65 (5.4)
high	104 (7.4)	115 (7.3)	4 (3.5)	10 (8.7)
missing	30 (2.2)	34 (2.1)		
**Education**[b]				
low	222 (16.2)	270 (17)	8 (3.0)	13 (4.8)
middle	502 (36.6)	588 (37.1)	34 (5.8)	31 (5.3)
high	294 (21.4)	324 (20)	6.2	5.6
missing	354 (25.8)	403 (25.4)		
**Days to surgery**				
< 48h	853 (62.2)	1012 (63.9)	57 (5.6)	49 (4.9)
> 48h	231 (16.8)	255 (16.1)	16 (6.1)	10 (3.9)
> 7 days	158 (11.5)	166 (10.5)	6 (3.6)	10 (6.0)
missing	130 (9.5)	152 (9.6)		
**Injured hand**				
Left	724 (52.8)	820 (51.7)	37 (4.5)	35 (4.3)
Right	644 (46.9)	761 (48)	42 (5.5)	41 (5.4)
missing	4 (0.3)	4 (0.3)		
**Number of fingers**				
Single	1201 (87.5)	1201 (75.8)	67 (5.6)	53 (4.4)
Multiple	171 (12.5)	384 (24.2)	13 (3.4)	23 (6.0)

[a] low income: disposable income per consumption unit below 60 % of median income for all. Middle income: income between low and high definition. High income: above double the median income.

[b] Education was defined as low: pre-high school, middle: high school, and high: post high school.
