# Supplementary material for: Risk factors for reoperation after flexor tendon repair: a registry study
Source: J Hand Surg Eur Vol. 2022 May 17;47(10):1071–6. doi: 10.1177/17531934221101563 (PMC9634328; doi:10.1177/17531934221101563)
Supplement: sj-pdf-3-jhs-10.1177_17531934221101563 - Supplemental material for Risk factors for reoperation after flexor tendon repair: a registry study [file sj-pdf-3-jhs-10.1177_17531934221101563.pdf]

Supplementary table 2. Demographic of variables from surgery and injury in the study cohort. Rate of rupture and tenolysis within each variable.

Variable	Total number of fingers (% of cohort)	Rupture rate (% fingers in variable)	Tenolysis rate (% fingers in variable)
**Injured tendon**			
FDP	620 (39)	17 (2.7)	19 (3.1)
FDP+partial FDS	236 (14.9)	9 (3.8)	14 (6.0)
FDP+FDS	458 (28.9)	30 (6.6)	33 (7.2)
FPL	223 (14.1)	23 (10.3)	10 (3.1)
missing	48 (3.0)		
**Injured finger**			
dig 1	223 (14.1)	23 (10.3)	10 (3.1)
dig 2	350 (22.1)	14 (4.0)	16 (4.6)
dig 3	223 (14.1)	14 (6.3)	8 (3.6)
dig 4	253 (16)	8 (3.2)	16 (6.3)
dig 5	534 (33.7)	21 (3.9)	27 (5.2)
missing	3 (0.1)		
**Injured digital nerves**			
none	970 (61.2)	49 (5.1)	44 (4.5)
one	497 (31.4)	27 (5.4)	23 (4.6)
both	114 (7.2)	4 (3.5)	9 (7.9)
missing	4 (0.3)		
**Core suture technique**			
Mod kessler	302 (19.1)	22 (7.3)	16 (5.3)
Tsuge/loop	821 (51.8)	44 (5.4)	43 (5.2)
reinsertion	95 (6)	4 (4.2)	0 (0.0)
criss cross	55 (3.5)	2 (3.6)	0 (0.0)
other	53 (3.3)	0 (0.0)	1 (1.9)
missing	259 (16.3)		
**Core suture material**			
braided polyester	553 (34.9)	27 (4.9)	34 (6.2)
Non-resorbable monofilament	156 (9.8)	10 (6.4)	4 (2.6)
Resorbable monofilament	95 (6)	5 (5.3)	1 (1.0)
Braided polyblend	478 (30.2)	29 (6.1)	19 (4.0)
other	24 (1.5)	0 (0.0)	1 (4.2)
missing	279 (17.6)		
**Core suture number**			
2	221 (13.9)	15 (6.8)	7 (3.2)
4	1033 (65.2)	52 (5.0)	50 (4.8)
other	38 (2.4)	2 (5.3)	1 (2.6)
missing	293 (18.5)		
**Core suture circumference**			
3-0	403 (25.4)	25 (6.2)	21 (5.2)
4-0	839 (52.9)	44 (5.2)	33 (3.9)
other	33 (2)	0 (0.0)	0 (0.0)

missing	310 (19.6)

FDP: Flexor digitorum profundus. FDS: Flexor digitorum superficialis. FPL: Flexor pollicis longus.
