# Supplementary material for: Risk factors for reoperation after flexor tendon repair: a registry study
Source: J Hand Surg Eur Vol. 2022 May 17;47(10):1071–6. doi: 10.1177/17531934221101563 (PMC9634328; doi:10.1177/17531934221101563)
Supplement: sj-pdf-4-jhs-10.1177_17531934221101563 - Supplemental material for Risk factors for reoperation after flexor tendon repair: a registry study [file sj-pdf-4-jhs-10.1177_17531934221101563.pdf]

**Supplementary table 3**. Information regarding individual variables and their unadjusted associations to rupture or tenolysis after flexor tendon repair in zone 1 and 2 in 1585 fingers.

| Variables | Unadjusted association to ruptur OR (CI 95%) | p-value | Unadjusted association to tenolysis OR (CI 95%) | p-value |
|---|---|---|---|---|
| Sex | | | | |
| Women | Reference | | Reference | |
| Men | 2.7 (1.4-5.3) | **0.004** | 0.7 (0.5-1.2) | 0.242 |
| Age | | | | |
| < 25 | Referencef | | Reference | |
| 25 - 50 | 5.2 (2.2-12.2) | **<0.001** | 1.1 (0.6-1.9) | 0.763 |
| > 50 | 5.8 (2.3-14.5) | **<0.001** | 1.4 (0.7-2.6) | 0.361 |
| Income[a] | | | | |
| low | 1.7 (0.5-5.4) | 0.4 | n.a | 0.994 |
| mid | 1.5 (0.5-4.1) | 0.464 | 0.6 | 0.143 |
| high | Reference | | Reference | |
| Education[b] | | | | |
| low | 0.5 (0.2-1.1) | 0.072 | 0.9 (0.4-1.8) | 0.686 |
| mid | 0.933 (0.5-1.6) | 0.811 | 0.9 (0.5-1.7) | 0.856 |
| high | Reference | ref | Reference | |
| Days to surgery | | | | |
| < 48h | Reference | | Reference | |
| > 48h | 0.9 (0.5-1.7) | 0.739 | 0.8 (0.4-1.6) | 0.494 |
| > 7 days | 0.6 (0.3-1.5) | 0.288 | 1.2 (0.6-2.5) | 0.557 |
| Injured hand | | | | |
| left | Reference | | Reference | |
| right | 1.2 (0.8-1.9) | 0.359 | 1.9 (0.8-2.0) | 0.300 |
| Injured tendon | | | | |
| FDP | Reference | | Reference | |
| FDP+partial FDS | 1.4 (0.6-3.2) | 0.416 | 2.0 (0.9-4.0) | 0.056 |
| FDP+FDS | 2.5 (1.4-4.6) | **0.003** | 2.5 (1.4-4.4) | **0.002** |
| FPL | 3.9 (2.0-7.5) | **<0.001** | 1.0 (0.4-2.5) | 0.940 |
| Number of fingers | | | | |
| single | 1.7 (0.9-3.1) | 0.091 | 1.4 (0.8-2.3) | 0.210 |
| multiple | Reference | | Reference | |
| Injured finger | | | | |
| dig 1 | 3.5 (1.5-8.0) | **0.003** | 0.5 (0.2-1.2) | 0.113 |
| dig 2 | 1.3 (0.5-3.1) | 0.589 | 0.7 (0.3-1.4) | 0.345 |
| dig 3 | 2.1 (0.8-5.0) | 0.113 | 0.6 (0.2-1.3) | 0.179 |
| dig 4 | Reference | | Reference | |
| dig 5 | 1.3 (0.5-3.1) | 0.593 | 0.8 (0.4-1.5) | 0.538 |
| Injured digital nerves | | | | |
| none | Reference | | Reference | |
| one | 1.1 (0.7-1.7) | 0.755 | 1.0 (0.6-1.7) | 0.924 |
| both | 0.7 (0.2-1.9) | 0.473 | 1.8 (0.9-3.8) | 0.121 |
| Core suture technique | | | | |
| Mod Kessler | 1.4 (0.8-2.4) | 0.226 | 1.0 (0.6-1.8) | 0.968 |

| | | | | |
|---|---|---|---|---|
| Tsuge/loop | Reference | | Reference | |
| reinsertion | 0.8 (0.3-2.2) | 0.635 | n.a | 0.996 |
| criss cross | 0.7 (0.2-2.8) | 0.582 | n.a | 0.997 |
| other | n.a | 0.997 | 0.3 (0.0-2.6) | 0.301 |
| Core suture material | | | | |
| braided polyester | 0.8 (0.5-1.4) | 0.404 | 1.6 (0.9-2.8) | 0.118 |
| Non-resorbable monofilament | 1.1 (0.5-2.2) | 0.877 | 0.6 (0.2-1.9) | 0.417 |
| Resorbable monofilament | 0.9 (0.3-2.3) | 0.762 | 0.3 (0.0-1.9) | 0.188 |
| Braided polyblend | Reference | | Reference | |
| other | n.a | 0.998 | 1.0 (0.1-8.2) | 0.963 |
| Core suture number | | | | |
| 2 | 1.4 (0.8-2.5) | 296 | 0.6 (0.3-1.4) | 0.281 |
| 4 | Reference | | Reference | |
| other | 1.0 (0.2-4.5) | 0.951 | 0.5 (0.1-3.9) | 0.536 |
| Core suture circumference | | | | |
| 3-0 | 1.2 (0.7-2.0) | 0.490 | 1.3 (0.8-2.4) | 0.303 |
| 4-0 | Reference | | Reference | |
| other | n.a | 0.998 | n.a | 0.998 |

[a] low income: disposable income per consumption unit below 60 % of median income for all. Middle income: income between low and high definition. High income: above double the median income.

[b] Education was defined as low: pre-high school, middle: high school, and high: post high school.

OR: odds ratio. CI: confidence interval. N.a: not applicable, due to zero observation with rupture or tenolysis. FDP: Flexor digitorum profundus. FDS: Flexor digitorum superficialis. FPL: Flexor pollicis longus.
